# Impact of Tumor Size on the Survival Benefit of Anatomic Versus Non-Anatomic Resection for Intrahepatic Cholangiocarcinoma

**DOI:** 10.1245/s10434-025-17270-4

**Published:** 2025-04-15

**Authors:** Jun Kawashima, Miho Akabane, Mujtaba Khalil, Selamawit Woldesenbet, Yutaka Endo, Kota Sahara, François Cauchy, Federico Aucejo, Hugo P. Marques, Rita Lopes, Andreia Rodriguea, Tom Hugh, Feng Shen, Shishir K. Maithel, Bas Groot Koerkamp, Irinel Popescu, Minoru Kitago, Matthew J. Weiss, Guillaume Martel, Carlo Pulitano, Luca Aldrighetti, George Poultsides, Andrea Ruzzente, Todd W. Bauer, Ana Gleisner, Itaru Endo, Roberto I. Troisi, Timothy M. Pawlik

**Affiliations:** 1 Department of Surgery, The Urban Meyer III and Shelley Meyer Chair for Cancer Research, Wexner Medical Center and James Comprehensive Cancer Center, The Ohio State University, Columbus, OH USA; 2 Department of Gastroenterological Surgery, Yokohama City University, Yokohama, Japan; 3 Department of Transplant Surgery, University of Rochester Medical Center, Rochester, NY USA; 4 Department of HPB Surgery and Liver Transplantation, Beaujon Hospital, Clichy, France; 5 Department of Hepato-Pancreato-Biliary and Liver Transplant Surgery, Cleveland Clinic Foundation, Digestive Diseases and Surgery Institute, Cleveland, OH USA; 6 Department of Surgery, Curry Cabral Hospital, Lisbon, Portugal; 7 Department of Surgery, The University of Sydney, Sydney, NSW Australia; 8 Department of Surgery, Eastern Hepatobiliary Surgery Hospital, Shanghai, China; 9 Division of Surgical Oncology, Winship Cancer Institution, Emory University, Atlanta, GA USA; 10 Department of Surgery, Erasmus University Medical Centre, Rotterdam, The Netherlands; 11 Department of Surgery, Fundeni Clinical Institute, Bucharest, Romania; 12 Department of Surgery, Keio University, Tokyo, Japan; 13 Department of Surgery, Cancer Institute, Northwell Health, New Hyde Park, NY USA; 14 Department of Surgery, University of Ottawa, Ottawa, ON Canada; 15 Department of Surgery, Royal Prince Alfred Hospital, Camperdown, NSW Australia; 16 Department of Surgery, San Raffaele Hospital, Milan, Italy; 17 Department of Surgery, Stanford University, Stanford, CA USA; 18 Division of General and Hepatobiliary Surgery, University of Verona, Verona, Italy; 19 Department of Surgery, University of Virginia, Charlottesville, VA USA; 20 Department of Surgery, University of Colorado Denver, Denver, CO USA; 21 Department of Clinical Medicine and Surgery, Federico II University, Naples, Italy

## Abstract

**Background:**

The role of anatomic resection (AR) versus non-anatomic resection (NAR) for intrahepatic cholangiocarcinoma (ICC) has not been thoroughly investigated. This study sought to define the impact of tumor size on the relative therapeutic benefit of AR versus NAR for ICC. Specifically, the study aimed to identify a threshold tumor size to define when AR rather than NAR may be warranted to achieve better survival outcomes for patients undergoing resection of ICC.

**Methods:**

Patients who underwent liver resection for ICC were identified from an international multi-institutional database. A multivariable Cox model with an interaction term was used to assess the relationship between tumor size and the survival impact of AR.

**Results:**

Among 969 patients, 506 (72.9 %) underwent AR, whereas 263 (27.1 %) had an NAR. Multivariable analysis demonstrated an interaction between tumor size and AR (hazard ratio [HR], 0.94; 95 % confidence interval [CI], 0.88–1.00; *p* = 0.045). A plot of the interaction demonstrated that AR was associated with improved outcomes for tumors size ≥4 cm. Among 257 (26.5 %) patients with tumors smaller than 4 cm, recurrence-free survival (RFS) did not differ between NAR and AR (3-year RFS: 65.2 % [95 % CI, 55.7–76.2] vs 58.1 % [95 % CI, 49.2–68.5]; *p* = 0.720). In contrast, among 712 (73.4 %) patients with tumors size ≥4 cm, AR was associated with improved RFS (3-year RFS: 34.7 % [95 % CI, 27.5–43.8] vs 44.9 % [95 % CI, 40.4–50.0]; *p* = 0.018).

**Conclusions:**

Anatomic resection was associated with improved RFS for ICC patients with tumors size ≥4 cm, indicating that tumor size may be a valuable criterion to determine the extent of liver resection for resectable ICC patients.

**Supplementary Information:**

The online version contains supplementary material available at 10.1245/s10434-025-17270-4.

Intrahepatic cholangiocarcinoma (ICC) is the second most common liver malignancy after hepatocellular carcinoma (HCC), with an increasing global incidence over the past three decades.^1,2^ Unfortunately, even after curative-intent surgery, the risk of recurrence remains high.^3,4^ In fact, up to 50 % to 80 % of patients experience recurrence within 2 years after surgery, with one in four patients having a recurrence within the first 6 months.^3,4^ The high incidence of recurrence contributes to a poor prognosis, with a median overall survival (OS) after resection ranging from 12 to 31 months.^4,5^ In turn, there has been interest in identifying surgical and systemic approaches that may lead to improved survival for patients with ICC.

Patients with ICC who are surgical candidates should undergo liver resection combined with locoregional lymph node dissection.^6–8^ Although the goal of hepatic resection is to achieve an R0 margin status, the extent of resection for ICC continues to be ill-defined.^6,9^ Similarly, the role of anatomic resection (AR) versus non-anatomic resection (NAR) in the treatment of patients with HCC has long been debated. Anatomic resection may be more effective due to removal of not only the tumor but also potential satellite lesions, micro-portal invasion, and intrahepatic metastases. In turn, the risk of recurrence after AR may be lower.^6,10^ In contrast, NAR preserves more of the liver parenchyma, which may be associated with lower perioperative morbidity and a reduced risk of inadequate future liver remnant (FLR) and postoperative liver insufficiency.^10^

Long-term outcomes comparing AR and NAR for patients with HCC remain inconsistent, with survival benefits of AR possibly limited to specific subgroups of patients.^10–12^ For example, several studies have noted that certain clinicopathologic factors such as tumor size, histologic differentiation, and microvascular invasion (MVI) may influence whether AR or NAR has a differential therapeutic benefit.^11,12^

In the context of ICC, we hypothesized that tumor biology may similarly play a role in determining the survival benefit of AR versus NAR for patients undergoing hepatic resection.^13^ Tumor size is a well-established predictor of survival and one of the few variables that can be reliably evaluated preoperatively. ^4^^,^^14^^,^^15^ Therefore, the objective of the current study was to define the impact of tumor size on the relative therapeutic benefit of AR versus NAR for ICC. Specifically, using a large, multi-institutional, international database, we sought to identify a threshold tumor size to define when AR rather than NAR may be warranted to achieve better survival outcomes for patients undergoing resection of ICC.

## Methods

### Data Source and Patient Selection

Patients who underwent curative-intent liver resection for solitary ICC tumor between 2000 and 2023 were identified from the International Intrahepatic Cholangiocarcinoma Study Group database.^4^ The study excluded patients who had extrahepatic metastasis, multiple tumors, direct invasion of contiguous organs, or an R1 resection margin. The study defined R1 resection as a tumor-free margin smaller than 1 mm.^16^ The study also excluded individuals with 90-day mortality, palliative surgery, or missing data on tumor size or surgical procedures. The study received approval from the institutional review board of each participating institution.

### Variables and Outcomes

Patient demographic and clinicopathologic variables included age, sex, American Society of Anesthesiologist (ASA) classification, region (i.e., Western country, Eastern country), year of surgery (i.e., 2000–2010, 2011–2023), receipt of neoadjuvant chemotherapy (NAC), cirrhosis, preoperative albumin-bilirubin (ALBI) score, preoperative carbohydrate antigen 19-9 (CA 19-9), type of surgery (i.e., NAR, AR), use of minimally invasive surgery (MIS), lymphadenectomy, tumor size, T category based on the American Joint Committee on Cancer (AJCC) eighth edition,^17^ nodal disease (i.e., N0 [negative], N1 [positive], Nx [not examined]), tumor-node-metastasis (TNM) stage based on the AJCC eighth edition,^17^ surgical margin, MVI, morphologic subtype (i.e., MF [mass-forming], IG [intraductal growth], PI [periductal infiltrating], MF+PI), tumor grade (i.e., well-, moderately, poorly differentiated; undifferentiated), perineural invasion (PNI), postoperative severe complication, and receipt of adjuvant chemotherapy. Liver resections were categorized as AR when they involved systematic removal of Couinaud segment(s) encompassing the tumor, including the tumor-bearing portal vein and the associated hepatic territory.^11,18^

The specific surgical techniques used to achieve AR were performed according to institutional and surgeon-specific protocols. Conversely, resections that did not adhere to the anatomic boundaries of liver segments were classified as NAR.^11,18^ Severity of postoperative complications was defined according to the Clavien-Dindo classification system (grades I to V). Severe complications were defined as Clavien-Dindo classification ≥III.^19^

The primary outcome was RFS, defined as the time elapsed between the date of liver resection and recurrence, confirmed either on biopsy or using evidence of a suspicious lesion on follow-up imaging. Additionally, the recurrence pattern was assessed and categorized as intrahepatic only, intra- and extrahepatic, extrahepatic only, or unknown. The secondary outcome was OS, defined as the interval between the date of resection and the date of death from any cause or the last follow-up visit.

After curative-intent hepatectomy, the patients were monitored for recurrence based on serum tumor markers and imaging, such as computed tomography (CT), magnetic resonance imaging (MRI), or both. The patients were followed once every 3 months during the first 3 years, once every 6 months during years 4 and 5, then annually thereafter.^4^

### Statistical Analysis

Descriptive statistics were presented as median values with interquartile ranges (IQRs) for continuous variables and as frequencies with percentages for categorical variables. Continuous variables were compared using the Mann-Whitney *U* or Kruskal-Wallis test, as appropriate. Categorical variables were compared with the chi-square test or Fisher’s exact test. Multiple imputations with chain equations (MICE) procedures were used to handle missing values.^20^ Survival was estimated using the Kaplan-Meier method and log-rank tests.

All potentially relevant variables were used to fit Cox proportional hazards regression models, with each variable tested individually. Any variable that had a significant association with RFS at a *p* value threshold lower than 0.1 was included in a multivariable Cox proportional hazards model. To evaluate the hypothesis that tumor size mediated the relationship between AR and survival, the model included an interaction term between tumor size and the type of resection (i.e., NAR or AR). Given the observed interaction, adjusted restricted cubic splines (RCS) for the hazard ratio (HR) of RFS were plotted to identify a threshold tumor size.^21^ This threshold was defined as the point below which tumor size was not associated with a significant difference in RFS, and above which AR was linked to a survival benefit.^21^ The variables used in the multivariable model were modeled using RCS with three pre-specified knots.^21^ The tumor size at which the RCS curves for the patients undergoing AR versus NAR began to diverge was identified.^21^

For additional analyses, the patients were stratified by TNM stage based on the AJCC eighth edition (i.e., stage I or II/IIIA/IIIB) and further analyzed according to the visualized threshold tumor size.^17^ The impact of AR was re-evaluated using Kaplan-Meier survival analysis and log-rank tests within each group. Furthermore, a sensitivity analysis was performed for the patients with 90-day mortality in the overall cohort to assess its potential impact on our findings. Statistical significance was set at an alpha of 0.05. All analyses were performed using R version 4.4.1 (R Project for Statistical Computing, Vienna, Austria).

## Results

### Patient Demographics

Among the 969 patients who met inclusion criteria, 549 (56.7 %) were male, and median age was 60 years (IQR, 52–69 years). A total of 374 (38.6 %) patients were ASA class >2, and 149 (15.4 %) patients had cirrhosis. A small subset of patients received NAC (*n* = 47, 4.9 %). The median preoperative ALBI score was –2.93 (IQR, –3.19 to –2.62), and median CA 19-9 level was 34 U/mL (IQR, 12–136 U/mL). Approximately half of patients (*n* = 485, 50.1 %) underwent lymphadenectomy, and a minority (*n* = 46, 4.7 %) underwent MIS. The median tumor size was 5.5 cm (IQR, 3.7–7.6 cm).

In terms of disease, 640 patients (66.0 %) had T1 tumors, 163 patients (16.8 %) had nodal metastasis (N1), and the majority (580 patients, 59.9 %) were classified as stage I. The median surgical margin was 5 mm (IQR, 2–10 mm), and 444 (45.8 %) patients had surgical margins less than 5 mm. A total of 259 (26.7 %) patients had MVI, with PI/MF+PI-type, poorly differentiated, or undifferentiated tumors, and PNI was present for 92 (9.5 %), 149 (15.4 %), and 165 (17 %) patients, respectively.

Postoperatively, 141 (14.6 %) patients experienced a severe complication, and 271 (28 %) patients received adjuvant chemotherapy (Table 1). To evaluate the impact of the time period, patient characteristics and RFS were compared among patients who underwent surgery from 2000 to 2010 versus individuals who underwent surgery from 2011 to 2023 (Table S1; Fig. S1).

**Table 1:** Clinicopathologic characteristics of the analytic cohort^a^

Characteristics	All patients	NAR	AR	*p* Value
	(*n* = 969) *n* (%)	(*n* = 263, 27.1 %) *n* (%)	(*n* = 706, 72.9 %) *n* (%)	
Median age: years (IQR)	60 (52–69)	58 (48–65)	62 (53–70)	<0.001
Male sex	549 (56.7)	188 (71.5)	361 (51.1)	<0.001
ASA classification >2	374 (38.6)	53 (20.2)	321 (45.5)	<0.001
Region: Eastern countries	405 (41.8)	188 (71.5)	217 (30.7)	<0.001
Year of surgery (2011–2023)	629 (64.9)	156 (59.3)	473 (67.0)	0.031
Neoadjuvant chemotherapy	47 (4.9)	2 (0.8)	45 (6.4)	0.001
Cirrhosis	149 (15.4)	69 (26.2)	80 (11.3)	<0.001
Median ALBI score (IQR)	–2.93 (–3.19 to –2.62)	–2.98 (–3.20 to –2.78)	–2.90 (–3.19 to –2.55)	0.005
Median CA19-9: U/mL (IQR)	34.0 (12.0–136.0)	27.0 (12.1–56.5)	38.5 (12.0–178.5)	0.005
Major hepatectomy	512 (52.8)		512 (72.5)	
Minimally invasive surgery	46 (4.7)	13 (4.9)	33 (4.7)	0.996
Lymphadenectomy	485 (50.1)	67 (25.5)	418 (59.2)	<0.001
Median tumor size: cm (IQR)	5.5 (3.7–7.6)	4.5 (3.0–6.0)	6.0 (4.0–8.1)	<0.001
Pathologic T category				<0.001
T1	640 (66.0)	200 (76.0)	440 (62.3)	
T2	141 (14.6)	22 (8.4)	119 (16.9)	
T3	188 (19.4)	41 (15.6)	147 (20.8)	
Pathologic N category				<0.001
N0	322 (33.2)	42 (16.0)	280 (39.7)	
N1	163 (16.8)	25 (9.5)	138 (19.5)	
Nx	484 (49.9)	196 (74.5)	288 (40.8)	
Pathologic TNM stage				<0.001
I	580 (59.9)	190 (72.2)	390 (55.2)	
II	100 (10.3)	18 (6.8)	82 (11.6)	
IIIA	126 (13.0)	30 (11.4)	96 (13.6)	
IIIB	163 (16.8)	25 (9.5)	138 (19.5)	
Median surgical margin: mm (IQR)	5.0 (2.0–10.0)	5.0 (2.0–10.0)	5.0 (2.0–10.00)	0.746
Surgical margin <5.0 mm	444 (45.8)	120 (45.6)	324 (45.9)	0.999
Microvascular invasion	259 (26.7)	45 (17.1)	214 (30.3)	<0.001
Morphologic type (PI/MF+PI)	92 (9.5)	4 (1.5)	88 (12.5)	<0.001
Grade, poorly differentiated/undifferentiated	149 (15.4)	20 (7.6)	129 (18.3)	<0.001
Perineural invasion	165 (17.0)	7 (2.7)	158 (22.4)	<0.001
Severe complication	141 (14.6)	17 (6.5)	124 (17.6)	<0.001
Adjuvant chemotherapy	271 (28.0)	26 (9.9)	245 (34.7)	<0.001

^a^Data are presented as median (IQR) for continuous measures and *n* (%) for categorical measures

*NAR* non-anatomic resection, *AR* anatomic resection, *ASA* American Society of Anesthesiologists, *ALBI* albumin-bilirubin, *CA19-9* carbohydrate antigen, *TNM* tumor-node-metastasis, *PI* periductal infiltrating, *MF* mass-forming

Among 969 patients, 263 (27.1 %) underwent NAR and 706 (72.9 %) underwent AR. Patients who underwent AR were more likely to be older (NAR vs AR: 58 years [IQR, 48–65 years] vs 62 years [IQR, 53–70 years]; *p* < 0.001), to have received NAC (*n* = 2 [0.8 %] vs *n* = 45 [6.4 %]; *p* = 0.001), and to have a higher ALBI score (–2.98 [IQR, –3.20 to –2.78] vs –2.90 [IQR, –3.19 to –2.55]; *p* = 0.005) as well as a higher CA 19-9 level (27.0 U/mL [IQR, 12.1–56.5 U/mL] vs 38.5 U/mL [IQR, 12.0–178.5 U/mL]; *p* = 0.005). In contrast, the AR patients were less likely to have cirrhosis (*n* = 69 [26.2 %] vs *n* = 80 [11.3 %]; *p* < 0.001). Patients who underwent AR versus NAR also were more likely to have larger tumors (4.5 cm [IQR, 3.0–6.0 cm] vs 6.0 cm [IQR, 4.0–8.1 cm]; *p* < 0.001) and to undergo lymphadenectomy (*n* = 67 [25.5 %] vs *n* = 418 [59.2 %]; *p* < 0.001).

On pathology, patients with T3 disease (*n* = 41 [15.6 %] vs *n* = 147 [20.8 %]; *p* < 0.001), N1 disease (*n* = 25 [9.5 %] vs *n* = 138 [19.5 %]; *p* < 0.001), or advanced-stage disease (stage II: *n* = 18 [6.8 %] vs *n* = 82 [11.6 %]; stage IIIA: *n* = 30 [11.4 %] vs *n* = 96 [13.6 %]; stage IIIB: *n* = 25 [9.5 %] vs *n* = 138 [19.5 %]; *p* < 0.001) were more likely to undergo AR. Moreover, patients who underwent AR were more likely to have MVI (*n* = 45 [17.1 %] vs *n* = 214 [30.3 %]; *p* < 0.001), PI/MF+PI (*n* = 4 [1.5 %] vs *n* = 88 [12.5 %]; *p* < 0.001), or poorly differentiated or undifferentiated tumors (*n* = 20 [7.6 %] vs *n* = 129 [18.3 %]; *p* < 0.001), as well as PNI (*n* = 7 [2.7 %] vs *n* = 158 [22.4 %]; *p* < 0.001). Notably, no differences were observed among patients who underwent NAR and AR regarding surgical margin (5 mm [IQR, 2–10 mm] vs 5 mm [IQR, 2–10 mm]; *p* = 0.746; Table 1).

### Interaction Between Tumor Size and Survival Benefit of AR

**Fig. 1:**
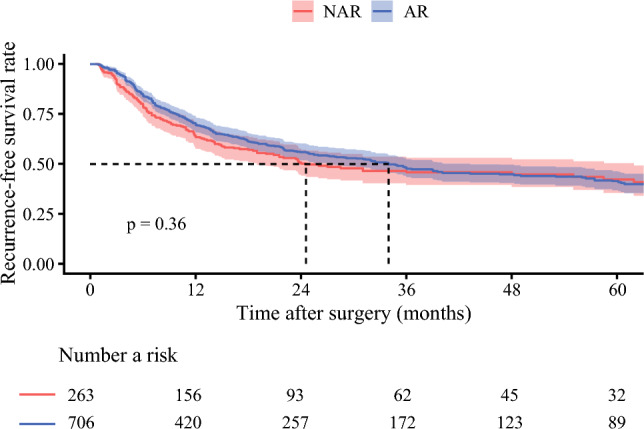
Kaplan-Meier curves comparing recurrence-free survival between patients in the entire cohort who underwent anatomic resection (AR) and those who had non-anatomic resection (NAR)

After a median follow-up period of 26.2 months (IQR, 13.9–35.8 months), the 3-year RFS was 45.8 % (95 % CI, 39.6–53.0 %) for patients who underwent NAR and 47.6 % (95 % CI, 43.4–52.1 %) for patients who underwent AR (*p* = 0.360; Fig. 1). The 5-year OS was 51.1 % (95 % CI, 43.8–59.6 %) for individuals who underwent NAR and 46.8 % (95 % CI, 42.2–51.8 %) for patients who had AR.

**Fig. 2:**
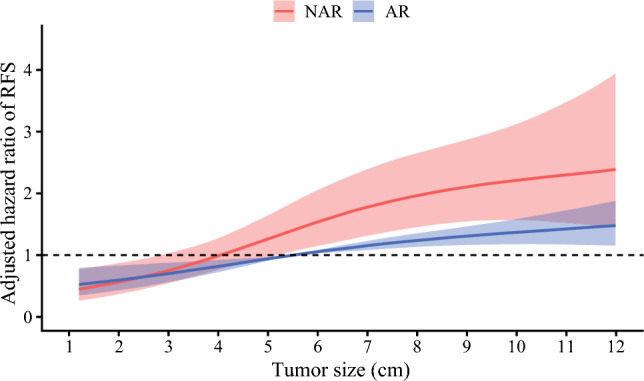
Plot illustrating the interaction between tumor size, surgical type (anatomic resection [AR] vs non-anatomic resection [NAR]), and the adjusted hazard of recurrence-free survival (RFS)

**Fig. 3:**
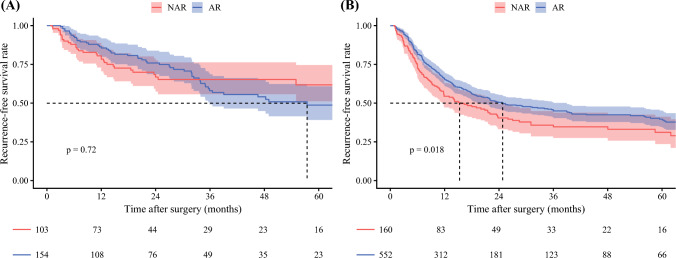
Kaplan-Meier curves comparing recurrence-free survival between patients who underwent anatomic resection (AR) and those who had non-anatomic resection (NAR), stratified by tumor size. **A** Patients with tumor smaller than 4 cm. **B** Patients with tumor size ≥4.0 cm

On multivariable Cox regression that included an interaction term between tumor size and type of surgery, age (HR, 0.98; 95 % CI, 0.98–0.99; *p* < 0.001), cirrhosis (HR, 1.30; 95 % CI, 1.01–1.67; *p* = 0.042), tumor size (HR, 1.15; 95 % CI, 1.09–1.21; *p* < 0.001), metastatic lymph node disease (HR, 1.59; 95 % CI, 1.20–2.09; *p* = 0.001), and MVI (HR, 1.41; 95 % CI, 1.11–1.78; *p* = 0.005) were independent preoperative predictors of recurrence, whereas AR was not associated with recurrence (HR, 1.13; 95 % CI, 0.73–1.73; *p* = 0.590). Notably, there was a modest interaction between tumor size and AR (HR, 0.94; 95 % CI, 0.88–1.00; *p* = 0.045; Table 2). A plot of the interaction between tumor size and type of surgery demonstrated that the survival curves for the patients undergoing NAR and those who had AR diverged beyond a tumor size of approximately 4 cm. These data suggested that patients were less likely to experience a survival benefit from AR below a tumor size threshold of 4 cm, whereas patients were more likely to benefit as tumor size increased beyond 4 cm (Fig. 2). Notably, among 257 (26.5 %) patients with tumor smaller than 4 cm, the 3-year RFS did not differ based on type of surgery (NAR vs AR: 65.2 % [95 % CI, 55.7–76.2 %] vs 58.1 % [95 % CI, 49.2–68.5 %]; *p* = 0.720; Fig. 3A). In contrast, among 712 (73.4 %) patients with tumor size ≥4 cm, AR was associated with improved 3-year RFS (NAR vs AR: 34.7 % [95 % CI, 27.5–43.8 %] vs 44.9 % [95 % CI, 40.4–50.0]; *p* = 0.018; Fig. 3B). These findings were confirmed in additional multivariable Cox models stratified by a tumor size threshold of 4 cm. Although AR was not associated with improved RFS among patients with tumors smaller than 4 cm (HR, 0.97; 95 % CI, 0.59–1.60; *p* = 0.898), AR remained independently associated with improved RFS among patients with tumors size ≥4 cm after adjustment for confounding factors (HR, 0.69; 95 % CI, 0.54–0.89; *p* = 0.004; Table S2).

**Table 2:** Uni- and multivariable COX regression analysis for recurrence

Variables	Reference	Univariate analysis		Multivariate analysis	
		HR 95 % CI	*p* Value^a^	HR 95 % CI	*p* Value^a^
Age		0.99 (0.98–0.99)	**0.001**	0.98 (0.98–0.99)	**<0.001**
Male sex	Female	1.10 (0.91–1.32)	0.334		
ASA classification >2	≤2	0.87 (0.72–1.05)	0.157		
Region (Eastern)	Western	1.01 (0.84–1.21)	0.940		
Year of surgery (2011–2023)	2000–2010	0.85 (0.70–1.02)	0.077	0.86 (0.70–1.04)	0.125
Neoadjuvant chemotherapy		1.09 (0.72–1.66)	0.676		
Cirrhosis		1.24 (0.98–1.57)	0.077	1.30 (1.01–1.67)	**0.042**
ALBI		1.08 (0.94–1.24)	0.294		
CA19-9		1.00 (1.00–1.00)	0.630		
Tumor size		1.08 (1.06–1.11)	**< 0.001**	1.15 (1.09–1.21)	**<0.001**
AR	NAR	0.91 (0.74–1.11)	0.362	1.13 (0.73–1.73)	0.590
Lymphadenectomy		1.05 (0.88–1.26)	0.563		
Pathologic T3	T1/T2	1.57 (1.26–1.94)	**< 0.001**	1.27 (0.99–1.63)	0.057
*Pathologic N category*					
N1	N0	1.79 (1.38–2.33)	**< 0.001**	1.59 (1.20–2.09)	**0.001**
Nx	N0	1.14 (0.93–1.40)	0.214	1.19 (0.95–1.49)	0.139
Surgical margin <5 mm	≥5 mm	1.13 (0.94–1.35)	0.195		
Microvascular invasion		1.52 (1.24–1.85)	**< 0.001**	1.41 (1.11–1.78)	**0.005**
Morphologic type (PI/MF+PI)	MF, IG	1.24 (0.93–1.66)	0.143		
Grade (poorly differentiated/undifferentiated)	Well-/moderately differentiated	1.25 (0.98–1.58)	0.068	1.18 (0.92–1.51)	0.198
Perineural invasion	Minor hepatectomy	1.41 (1.12–1.78)	**0.003**	1.26 (0.96–1.64)	0.090
Adjuvant chemotherapy		1.04 (0.85–1.26)	0.708		
Tumor size × AR	NAR			0.94 (0.88–1.00)	**0.045**

*HR* hazard ratio, *CI* confidence interval, *ASA* American Society of Anesthesiologists, *ALBI* albumin-bilirubin, *CA19-9* carbohydrate antigen, *AR* anatomic resection, *NAR* non-anatomic resection, *PI* periductal infiltrating, *Nx* not examined, *MF* mass-forming, *IG*  intraductal growth

^a^Bold font signifies *p* value <0.05

The recurrence pattern was evaluated based on a tumor size threshold of 4 cm. Among 93 patients with tumors smaller than 4 cm who experienced recurrence, no difference was noted between patients who underwent NAR and those who had AR (Table S3). In contrast, among 377 patients with tumors size ≥4 cm who experienced recurrence, individuals who underwent NAR were more likely to have an intrahepatic-only recurrence pattern (NAR: n = 74 [74.0 %] vs AR: *n* = 135 [48.7 %]; *p* < 0.001; Table S4).

On OS analysis, AR and NAR did not differ among patients with tumors either size ≥4 cm (5-year OS: NAR vs AR, 40.9 % [95 % CI, 32.3–51.9 %] vs 43.8 % [95 % CI, 38.8–49.4 %]; *p* = 0.330) or smaller than 4 cm (5-year OS: NAR vs AR, 69.3 % [95 % CI, 58.2–82.5 %] vs 57.8 % [95 % CI, 48.1–69.5 %]; *p* = 0.280; Fig. S2).

### Additional Analysis Stratified by TNM Stage

**Fig. 4:**
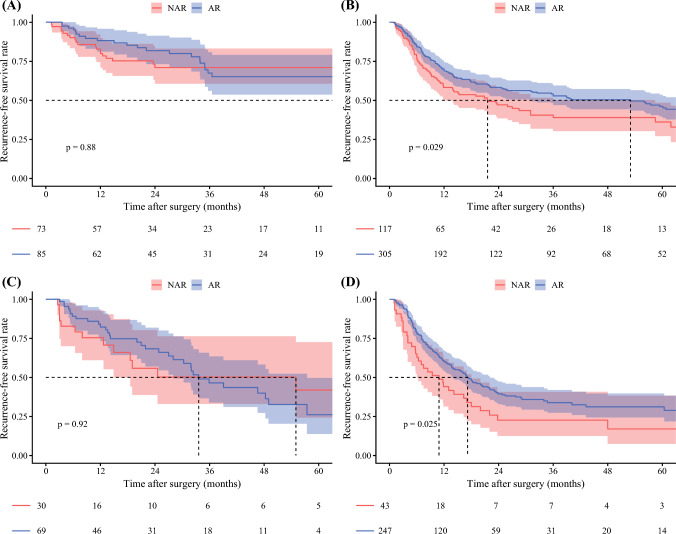
Kaplan-Meier curves comparing recurrence-free survival between patients who underwent anatomic resection (AR) and those who had non-anatomic resection (NAR), stratified by stage and tumor size. **A** Stage I disease with tumor smaller than 4 cm. **B** Stage I disease with tumor size ≥4 cm. **C** Stage II/III disease with tumor smaller than 4 cm. **D** Stage II/III disease with tumor size ≥4 cm

An additional analysis stratified the cohort into early-stage (TNM stage I) and advanced-stage (TNM stage II, IIIA, or IIIB) groups. Among early-stage patients with tumors smaller than 4 cm (*n* = 158), no difference in RFS was observed among individuals who underwent AR and those who had NAR (NAR vs AR: 3-year RFS, 71.0 % [95 % CI, 60.6–83.2 %] vs 67.4 % [95 % CI, 56.3–80.9]; *p* = 0.880; Fig. 4A). Notably, among patients with tumors size ≥4 cm (*n* = 422), AR was associated with better RFS (NAR vs AR: 3-year RFS, 39.0 % [95 % CI, 30.3–50.2 %] vs 52.8 % [95 % CI, 47.0–59.4 %]; *p* = 0.029; Fig. 4B). A similar trend was observed among advanced-stage patients. Among patients with tumors smaller than 4 cm (*n* = 99), no difference in RFS was noted relative to the surgical approach (NAR vs AR: 3-year RFS, 50.2 % [95 % CI, 33.1–76.3 %] vs 46.4 % [95 % CI, 34.0–63.5 %]; *p* = 0.920; Fig. 4C). In contrast, among patients with tumors size ≥4 cm (*n* = 290), AR was associated with improvement in RFS (NAR vs AR: 3-year RFS, 22.6 % [95 % CI, 12.6–40.7 %] vs 33.9 % [95 % CI, 27.4–41.9 %]; *p* = 0.025; Fig. 4D).

### Additional Analysis Including Patients With 90-Day Mortality

A sensitivity analysis of 41 patients with 90-day mortality in the overall cohort (*n* = 1010) was performed. Among 263 (26.0 %) patients with tumor smaller than 4 cm, the 3-year RFS remained similar between patients who underwent NAR and those who had AR (65.3 % [95 % CI, 55.9–76.3 %] vs 58.1 % [95 % CI, 49.2–68.5 %]; *p* = 0.710). In contrast, among 747 (74.0 %) patients with tumor size ≥4 cm, AR remained associated with improved 3-year RFS (NAR vs AR: 34.9 % [95 % CI, 27.7–44.1 %] vs 44.9 % [95 % CI, 40.4–49.9 %]; *p* = 0.021; Fig. S3).

## Discussion

Although liver resection with an R0 margin remains the mainstay of treatment for ICC, international guidelines offer no clear recommendations on whether AR or NAR is preferred.^7,8,22,23^ The European Society for Medical Oncology (ESMO) and the European Network for the Study of Cholangiocarcinoma (ENS-CCA) do not specify the necessity of AR over NAR, whereas the 2024 National Comprehensive Cancer Network (NCCN) guidelines consider both approaches acceptable as long as negative margins are achieved.^7,22,23^ In contrast, the European Association for the Study of the Liver (EASL) and the International Liver Cancer Association (ILCA) recommend AR as the preferred treatment.^8^ These conflicting recommendations can be attributed to the absence of randomized controlled trials comparing AR and NAR and inconsistent results from retrospective studies.^6^ For example, a single-institution study with approximately 700 ICC patients noted that AR was associated with better RFS and OS than NAR.^24^ In contrast, a multi-institutional study from China reported no difference in RFS between AR and NAR (median RFS, 20 vs 17 months, respectively; *p* = 0.340).^25^

Although the explanation for these disparate data is likely multifactorial, these discrepancies may result from the fact that subgroups of patients most likely to benefit from AR have not been clearly identified.^13^ Therefore, the current study was important because we identified a notable relationship between larger tumor size and a survival benefit of AR for patients undergoing curative-intent resection for ICC, using a large international multi-institutional database. Furthermore, the use of RCS analysis demonstrated that AR was not associated with a survival benefit for patients with tumors smaller than 4 cm.

In contrast, a survival benefit from AR was observed among patients with tumor size ≥4 cm. Notably, this survival benefit of AR for tumors larger beyond 4 cm was consistent regardless of pathologic TNM stage. Thus, AR conferred a survival advantage for tumors size ≥4 cm, whereas no survival benefit existed for tumors smaller than 4 cm regardless of tumor stage. These findings underscore the applicability of a 4 cm threshold to assist surgeons in deciding whether to perform AR or NAR for ICC. Although extensively studied in HCC, the impact of AR versus NAR on oncologic outcomes related to ICC has not been examined.^9–13^ The concept of AR, introduced by Makuuchi et al.^26^ in 1985, involves removing the hepatic segment or subsegment that includes the tumor-bearing portal tributaries, as well as major branches of the portal vein and hepatic artery.^26^ Theoretically, AR may provide superior locoregional control by removal of the entire tumor burden, including any micro-portal invasion and intra-hepatic metastases.^10,27^ On the other hand, NAR may better preserve healthy liver tissue, reducing the risk of postoperative liver insufficiency.^28^ In addition, NAR of the primary ICC tumor may expand eligibility for future surgical treatment in cases of resectable intrahepatic recurrence, for which repeat liver resection treatment has been suggested to improve survival outcomes.^29,30^ As such, the ideal surgical approach to hepatic resection of ICC should optimize locoregional control yet preserve as much non-tumorous hepatic parenchyma in the FLR as possible.^10^ The effectiveness of locoregional control may vary depending on tumor biology, suggesting that whereas some patients may benefit from AR, others might achieve comparable outcomes with R0 resection alone.^13^ In the field of HCC, several investigators have demonstrated that the survival benefit of AR is linked to prognostic factors such as tumor size and vascular invasion.^11,12,31,32^ Similarly, the current study demonstrated that ICC patients with larger tumors derived a survival benefit from AR, whereas AR was not associated with a survival benefit for patients with a tumor smaller than 4 cm. In particular, among patients with tumors size ≥4 cm, those who underwent NAR were more likely to experience intrahepatic-only recurrence. These data suggested that AR may play a crucial role in controlling microscopic intrahepatic dissemination in larger tumors, potentially by removing undetectable satellite lesions or by microscopic vascular invasion.

Among ICC patients who undergo curative-intent surgery, tumor size is a well-established prognostic factor.^4,14,15,33,34^ For instance, a meta-analysis of 4765 patients who underwent surgery for ICC noted that larger tumor size was associated with worse overall OS.^33^ Additionally, Tsilimigras et al.^4^ reported a 12 % increased risk of early recurrence for every 1-cm increase in tumor size. Consistent with these findings, the current study also demonstrated that larger tumor size was associated with worse long-term outcomes, reinforcing the idea that tumor size serves as an indicator of oncologic burden for ICC patients after liver resection. However, research on how tumor size interacts with type of surgery has been limited, particularly regarding whether tumor size can stratify patients based on their likelihood of benefiting from AR. This point is a critical consideration because decisions regarding AR or NAR typically are made preoperatively, and tumor size is one of the few prognostic factors that can be estimated before surgery.^24^

Importantly, the current study demonstrated that tumor size markedly influenced the survival benefit associated with AR for ICC patients. By using RCS analysis, we identified a critical tumor size threshold of 4 cm, beyond which AR was associated with a survival benefit. Supporting the findings, a single-institution retrospective study indicated that patients with TNM stage IB, characterized by a solitary tumor larger than 5 cm, experienced a survival benefit from AR.^24^ In contrast, patients with TNM stage IA, defined as a solitary tumor of 5 cm or smaller, did not have a survival benefit from AR.^24^ These findings underscore the importance of incorporating tumor size into the preoperative decision-making process when AR versus NAR is considered for ICC patients.

To account for important pathologic factors such as lymph node metastasis and vascular invasion, we further stratified the patients into early and advanced stages based on the eighth AJCC staging system.^17^ The patients with stage II or III disease, characterized by vascular invasion, visceral peritoneum perforation, or lymph node metastasis, were classified as having advanced-stage disease, whereas those who had stage I disease without these factors were categorized as having early-stage disease.^17,24,35^

Among patients with ICC, lymph node metastasis and MVI are established prognostic factors.^4,33,36^ For example, a recent systematic review demonstrated that ICC patients with lymph node metastasis or MVI were respectively 57 % or 42 % more likely to experience early recurrence.^36^ Consistent with previous studies, these factors were identified as independent predictors of recurrence in the current study, suggesting that these factors need to be considered when the type of surgery is being determined.^24^ However, unlike tumor morphology, these pathologic factors are difficult to assess preoperatively.^24^

Notably, in the current study, AR did not confer a survival advantage for tumors smaller than 4 cm irrespective of other pathologic features. In contrast, for tumors size ≥4 cm, AR was associated with a survival benefit, even when other adverse pathologic factors were considered. These findings suggest that a 4-cm tumor size threshold is important in determining the survival benefit of AR for ICC, independently of other prognostic factors included in the TNM staging system. Consequently, tumor size should be a primary consideration in surgical planning for ICC regardless of advanced pathologic features.

In the current study, the incidence of severe complications was higher among the patients undergoing AR versus NAR (17.6 % vs 6.5 %; *p* < 0.001). As such, AR should be reserved for cases for which the oncologic benefits are justified. We found AR to be associated with prolonged RFS, particularly for tumors size ≥4 cm, suggesting a potential oncologic advantage. However, this benefit did not translate into a survival benefit in terms of OS, regardless of tumor size. The lack of an OS benefit may be due to the availability of salvage therapies after recurrence, as well as the relatively moderate follow-up period duration (median, 26.2 months [IQR, 13.9–35.8 months]) and sample size, which may have reduced the statistical power to detect OS differences. Given the increased morbidity associated with AR and the ongoing controversy regarding its long-term benefit, even among patients with tumors size ≥4 cm, surgical decision-making should be individualized, particularly for high-risk patients, such as the elderly or individuals with major comorbidities or impaired liver function.

Several limitations should be considered when the results of the current study are interpreted. Although the multi-institutional nature of the database was a strength, heterogeneity in patient selection and surgical techniques likely existed across participating centers. In particular, variability in the criteria used by institutions and surgeons to determine the extent of liver resection may have introduced selection bias. In the current study, because AR was performed according to institutional and surgeon-specific protocols, the method used to delineate the anatomic boundaries of resection may have varied among institutions. Intraoperative techniques such as indocyanine green fluorescence imaging, intraoperative ultrasonography, and three-dimensional navigation were not standardized across centers, which may have led to variability in defining resection planes, possibly influencing the oncologic outcomes. Furthermore, due to the rarity of ICC, a large international multi-institutional database spanning multiple decades was required to achieve a sufficient sample size.

To account for potential temporal biases, the time period (2000–2010 vs 2011–2023) was included as a covariate in the multivariable model. Although this approach allowed for adjustment of major temporal variations, the influence of unmeasured confounders related to evolving clinical practices could not be fully excluded. In addition, although the current study identified a tumor size threshold of 4 cm using a large international multi-institutional database, the external validity of the findings requires further evaluation. A prospective study with standardized surgical techniques and perioperative management is needed to validate our findings.

In summary, AR was not associated with a survival benefit for the patients with an ICC tumor size smaller than 4 cm. In contrast, the patients with tumors 4 cm or larger experienced a survival benefit with AR after curative-intent hepatic resection of ICC. Notably, this 4 cm tumor size threshold was applicable to both patients with early and those with advanced-stage features. These findings suggest that tumor size may be a valuable criterion in surgical decision-making relative to AR versus NAR for ICC patients.

## Supplementary Information

Below is the link to the electronic supplementary material.

Supplementary file1 (DOCX 572 kb)

## References

[1] Wu L, Tsilimigras DI, Paredes AZ, et al. Trends in the incidence, treatment and outcomes of patients with intrahepatic cholangiocarcinoma in the USA: facility type is associated with margin status, use of lymphadenectomy, and overall survival. *World J Surg*. 2019;43:1777–87. 10.1007/s00268-019-04966-4. 30820734 10.1007/s00268-019-04966-4

[2] Singal AK, Vauthey J-N, Grady JJ, Stroehlein JR. Intra-hepatic cholangiocarcinoma—frequency and demographic patterns: thirty-year data from the M.D. Anderson cancer center. *J Cancer Res Clin Oncol*. 2011;137(7):1071–8. 10.1007/s00432-010-0971-z. 21207060 10.1007/s00432-010-0971-z PMC11827973

[3] Zhang XF, Beal EW, Bagante F, et al. Early versus late recurrence of intrahepatic cholangiocarcinoma after resection with curative intent. *Br J Surg*. 2018;105:848–56. 10.1002/bjs.10676. 29193010 10.1002/bjs.10676

[4] Tsilimigras DI, Sahara K, Wu L, et al. Very early recurrence after liver resection for intrahepatic cholangiocarcinoma: considering alternative treatment approaches. *JAMA Surg*. 2020;155:823–31. 10.1001/jamasurg.2020.1973. 32639548 10.1001/jamasurg.2020.1973 PMC7344787

[5] Kim DH, Choi DW, Choi SH, Heo JS, Kow AW. Is there a role for systematic hepatic pedicle lymphadenectomy in intrahepatic cholangiocarcinoma? a review of 17 years of experience in a tertiary institution. *Surgery*. 2015;157:666–75. 10.1016/j.surg.2014.11.006. 25682172 10.1016/j.surg.2014.11.006

[6] Berardi G, Risi L, Muttillo EM, et al. Anatomic versus non-anatomic liver resection for intrahepatic cholangiocarcinoma: a systematic review and patient-level meta-analysis. *Ann Surg Oncol*. 2024;31:9170–82. 10.1245/s10434-024-16121-y. 39251512 10.1245/s10434-024-16121-y

[7] NCCN Guidelines Version 3.2024 Intrahepatic Cholangiocarcinoma. 2024. Retrieved December 1, 2024 at https://www.nccn.org/professionals/physician_gls/pdf/btc.pdf.

[8] European association for the study of the liver. EASL-ILCA Clinical Practice Guidelines on the management of intrahepatic cholangiocarcinoma [published correction appears in *J Hepatol.* 2023;79:1342. 10.1016/j.jhep.2023.09.006]. *J Hepatol*. 2023;79:181–208. 10.1016/j.jhep.2023.03.010 10.1016/j.jhep.2023.03.010 37084797

[9] Cloyd JM, Ejaz A, Pawlik TM. The landmark series: intrahepatic cholangiocarcinoma. *Ann Surg Oncol*. 2020;27:2859–65. 10.1245/s10434-020-08621-4. 32419038 10.1245/s10434-020-08621-4

[10] Moris D, Tsilimigras DI, Kostakis ID, et al. Anatomic versus non-anatomic resection for hepatocellular carcinoma: a systematic review and meta-analysis. *Eur J Surg Oncol*. 2018;44:927–38. 10.1016/j.ejso.2018.04.018. 29751946 10.1016/j.ejso.2018.04.018

[11] Shin SW, Kim T-S, Ahn KS, Kim YH, Kang KJ. Effect of anatomical liver resection for hepatocellular carcinoma: a systematic review and meta-analysis. *Int J Surg*. 2023. 10.1097/JS9.0000000000000503. 37247010 10.1097/JS9.0000000000000503 PMC10498869

[12] Hidaka M, Eguchi S, Okuda K, et al. Impact of anatomical resection for hepatocellular carcinoma with microportal invasion (vp1): a multi-institutional study by the kyushu study group of liver surgery. *Ann Surg*. 2020;271:339–46. 10.1097/SLA.0000000000002981. 30048313 10.1097/SLA.0000000000002981

[13] Ruff SM, Pawlik TM. Clinical management of intrahepatic cholangiocarcinoma: surgical approaches and systemic therapies. *Front Oncol*. 2024. 10.3389/fonc.2024.1321683. 38344197 10.3389/fonc.2024.1321683 PMC10853998

[14] Kawashima J, Sahara K, Shen F, et al. Predicting risk of recurrence after resection of stage I intrahepatic cholangiocarcinoma. *J Gastrointest Surg*. 2024;28:18–25. 10.1016/j.gassur.2023.10.002. 38353070 10.1016/j.gassur.2023.10.002

[15] Bagante F, Spolverato G, Merath K, et al. Intrahepatic cholangiocarcinoma tumor burden: a classification and regression tree model to define prognostic groups after resection. *Surgery*. 2019;166:983–90. 10.1016/j.surg.2019.06.005. 31326191 10.1016/j.surg.2019.06.005

[16] Endo Y, Sasaki K, Moazzam Z, et al. Higher tumor burden status dictates the impact of surgical margin status on overall survival in patients undergoing resection of intrahepatic cholangiocarcinoma. *Ann Surg Oncol*. 2023;30:2023–32. 10.1245/s10434-022-12803-7. 36396868 10.1245/s10434-022-12803-7

[17] Amin MB, Greene FL, Edge SB, et al. The eighth-edition AJCC cancer staging manual: continuing to build a bridge from a population-based to a more “personalized” approach to cancer staging. *CA Cancer J Clin*. 2017;67:93–9. 10.3322/caac.21388. 28094848 10.3322/caac.21388

[18] Cucchetti A, Cescon M, Ercolani G, Bigonzi E, Torzilli G, Pinna AD. A comprehensive meta-regression analysis on outcome of anatomic resection versus nonanatomic resection for hepatocellular carcinoma. *Ann Surg Oncol*. 2012;19:3697–705. 10.1245/s10434-012-2450-z. 22722807 10.1245/s10434-012-2450-z

[19] Dindo D, Demartines N, Clavien PA. Classification of surgical complications: a new proposal with evaluation in a cohort of 6336 patients and results of a survey. *Ann Surg*. 2004;240:205–13. 10.1097/01.sla.0000133083.54934.ae. 15273542 10.1097/01.sla.0000133083.54934.ae PMC1360123

[20] van Buuren S, Boshuizen HC, Knook DL. Multiple imputation of missing blood pressure covariates in survival analysis. *Stat Med*. 1999;18:681–94. 10204197 10.1002/(sici)1097-0258(19990330)18:6<681::aid-sim71>3.0.co;2-r

[21] Raman V, Jawitz OK, Farrow NE, et al. The relationship between lymph node ratio and survival benefit with adjuvant chemotherapy in node-positive esophageal adenocarcinoma. *Ann Surg*. 2022;275:e562–7. 10.1097/SLA.0000000000004150. 32649467 10.1097/SLA.0000000000004150 PMC7790855

[22] Vogel A, Bridgewater J, Edeline J, et al. Biliary tract cancer: ESMO clinical practice guideline for diagnosis, treatment, and follow-up. *Ann Oncol*. 2023;34:127–40. 10.1016/j.annonc.2022.10.506. 36372281 10.1016/j.annonc.2022.10.506

[23] Banales JM, Marin JJG, Lamarca A, et al. Cholangiocarcinoma 2020: the next horizon in mechanisms and management. *Nat Rev Gastroenterol Hepatol*. 2020;17:557–88. 10.1038/s41575-020-0310-z. 32606456 10.1038/s41575-020-0310-z PMC7447603

[24] Si A, Li J, Yang Z, et al. Impact of anatomical versus non-anatomical liver resection on short- and long-term outcomes for patients with intrahepatic cholangiocarcinoma. *Ann Surg Oncol*. 2019;26:1841–50. 10.1245/s10434-019-07260-8. 30843164 10.1245/s10434-019-07260-8

[25] Ke Q, Wang L, Lin Z, Liu H, Lou J, Zheng S, Bi X, Wang J, Guo W, Li F, Wang J, Zheng Y, Li J, Cheng S, Zhou W, Liu J, Zeng Y. Anatomic versus non-anatomic resection for early-stage intrahepatic cholangiocarcinoma: a propensity score matching and stabilized inverse probability of treatment weighting analysis. *BMC Cancer*. 2023. 10.1186/s12885-023-11341-z. 37697239 10.1186/s12885-023-11341-z PMC10496223

[26] Makuuchi M, Hasegawa H, Yamazaki S. Ultrasonically guided subsegmentectomy. *Surg Gynecol Obstet*. 1985;161:346–50. 2996162

[27] Regimbeau JM, Kianmanesh R, Farges O, Dondero F, Sauvanet A, Belghiti J. Extent of liver resection influences the outcome in patients with cirrhosis and small hepatocellular carcinoma. *Surgery*. 2002;131:311–7. 10.1067/msy.2002.121892. 11894036 10.1067/msy.2002.121892

[28] Moris D, Ronnekleiv-Kelly S, Rahnemai-Azar AA, et al. Parenchymal-sparing versus anatomic liver resection for colorectal liver metastases: a systematic review. *J Gastrointest Surg*. 2017;21:1076–85. 10.1007/s11605-017-3397-y. 28364212 10.1007/s11605-017-3397-y

[29] Ramouz A, Ali-Hasan-Al-Saegh S, Shafiei S, Fakour S, Khajeh E, Majlesara A, et al. Repeat liver resection for recurrent intrahepatic cholangiocarcinoma: meta-analysis. *Br J Surg*. 2022;109:580–7. 35482020 10.1093/bjs/znac075

[30] Tsilimigras DI, Endo Y, Guglielmi A, et al. Recurrent intrahepatic cholangiocarcinoma: a 10-point score to predict post-recurrence survival and guide treatment of recurrence. *Ann Surg Oncol*. 2024;31:4427–35. 10.1245/s10434-024-15210-2. 38520582 10.1245/s10434-024-15210-2

[31] Eguchi S, Kanematsu T, Arii S, et al. Comparison of the outcomes between an anatomical subsegmentectomy and a non-anatomical minor hepatectomy for single hepatocellular carcinomas based on a Japanese nationwide survey. *Surgery*. 2008;143:469–75. 10.1016/j.surg.2007.12.003. 18374043 10.1016/j.surg.2007.12.003

[32] Zhao H, Chen C, Gu S, et al. Anatomical versus non-anatomical resection for solitary hepatocellular carcinoma without macroscopic vascular invasion: a propensity score-matching analysis. *J Gastroenterol Hepatol*. 2017;32:870–8. 10.1111/jgh.13603. 27671209 10.1111/jgh.13603

[33] Mavros MN, Economopoulos KP, Alexiou VG, Pawlik TM. Treatment and prognosis for patients with intrahepatic cholangiocarcinoma: systematic review and meta-analysis. *JAMA Surg*. 2014;149:565–74. 10.1001/jamasurg.2013.5137. 24718873 10.1001/jamasurg.2013.5137

[34] Tsilimigras DI, Hyer JM, Paredes AZ, et al. Tumor burden dictates prognosis among patients undergoing resection of intrahepatic cholangiocarcinoma: a tool to guide post-resection adjuvant chemotherapy? *Ann Surg Oncol*. 2021;28:1970–8. 10.1245/s10434-020-09393-7. 33259043 10.1245/s10434-020-09393-7

[35] Zhang X-F, Xue F, He J, et al. Proposed modification of the eighth edition of the AJCC staging system for intrahepatic cholangiocarcinoma. *HPB*. 2021;23(9):1456–66. 10.1016/j.hpb.2021.02.009. 33814298 10.1016/j.hpb.2021.02.009

[36] Choi WJ, Williams PJ, Claasen MPAW, et al. Systematic review and meta-analysis of prognostic factors for early recurrence in intrahepatic cholangiocarcinoma after curative-intent resection. *Ann Surg Oncol*. 2022. 10.1245/s10434-022-11463-x. 35181812 10.1245/s10434-022-11463-x

