# Supplementary material for: Impact of Tumor Size on the Survival Benefit of Anatomic Versus Non-Anatomic Resection for Intrahepatic Cholangiocarcinoma
Source: Ann Surg Oncol. 2025 Apr 15;32(8):5637–47. doi: 10.1245/s10434-025-17270-4 (PMC12222373; doi:10.1245/s10434-025-17270-4)
Supplement: Supplementary file 1 — Supplementary file1 (DOCX 572 kb) [file 10434_2025_17270_MOESM1_ESM.docx]

**Supplementary Figure 1**: Kaplan-Meier curves comparing recurrence-free survival between patients who underwent surgery from 2000 to 2010 and individuals who underwent surgery from 2011 to 2023 in the entire cohort.


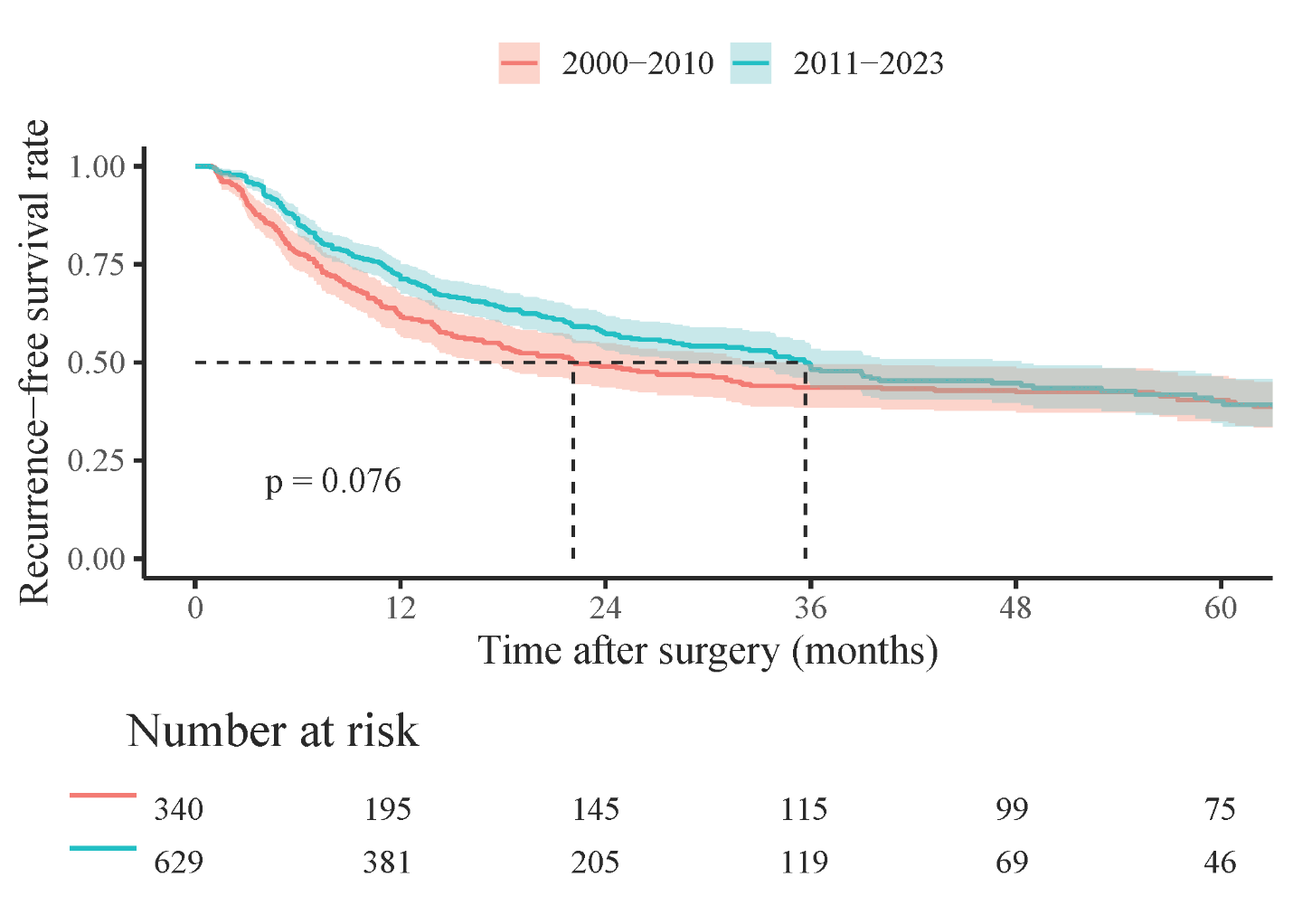


**Supplementary Figure 2**: Kaplan-Meier curves comparing overall survival between patients who underwent anatomic resection (AR) and non-anatomic resection (NAR), stratified by tumor size: (A) patients with tumor size < 4.0 cm, and (B) patients with tumor size ≥ 4.0 cm.


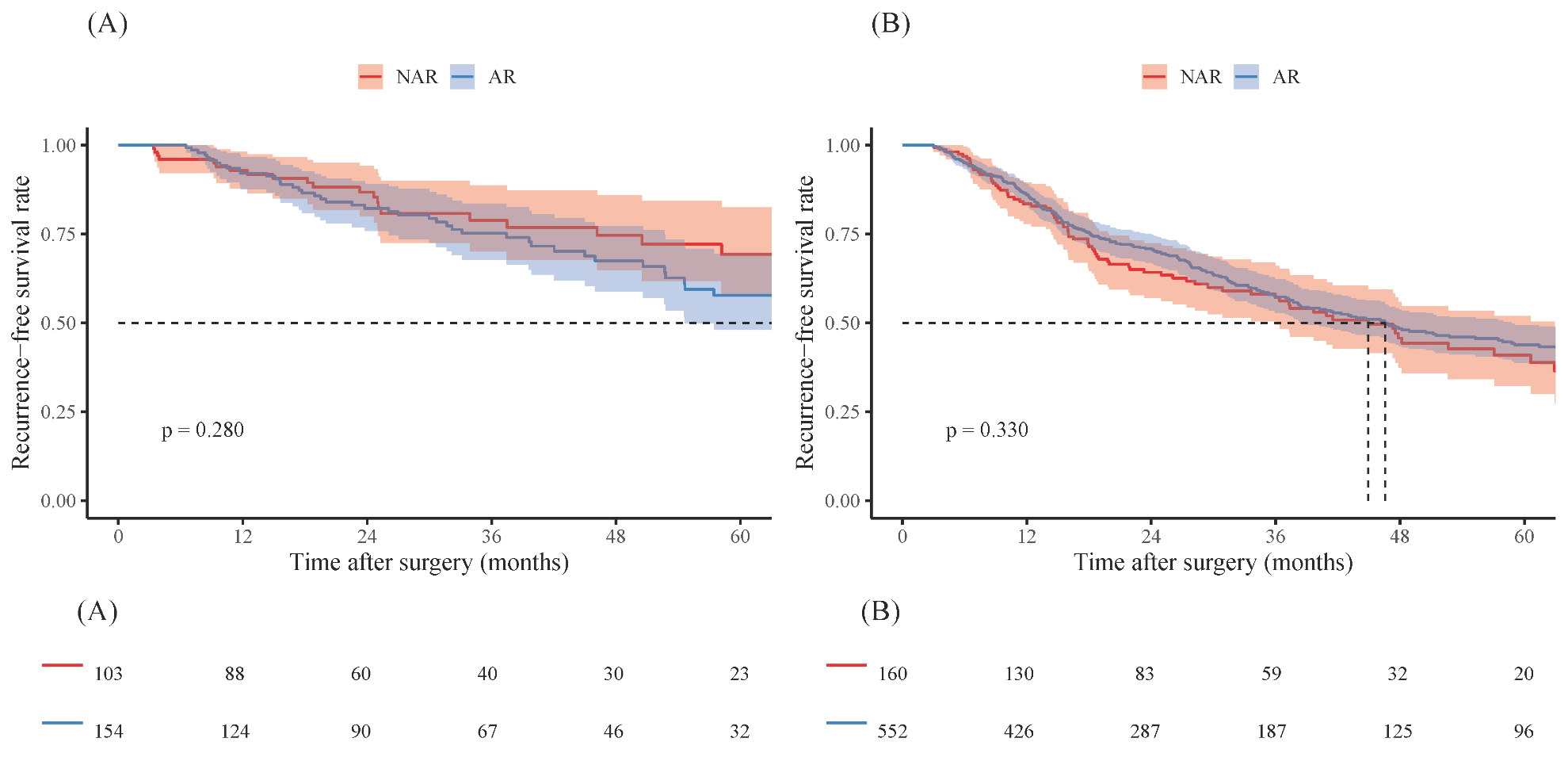


**Supplementary Figure 3**: Kaplan-Meier curves comparing recurrence-free survival between patients who underwent anatomic resection (AR) and non-anatomic resection (NAR), stratified by tumor size: (A) patients with tumor size < 4.0 cm, and (B) patients with tumor size ≥ 4.0 cm, including those who experienced 90-day mortality.


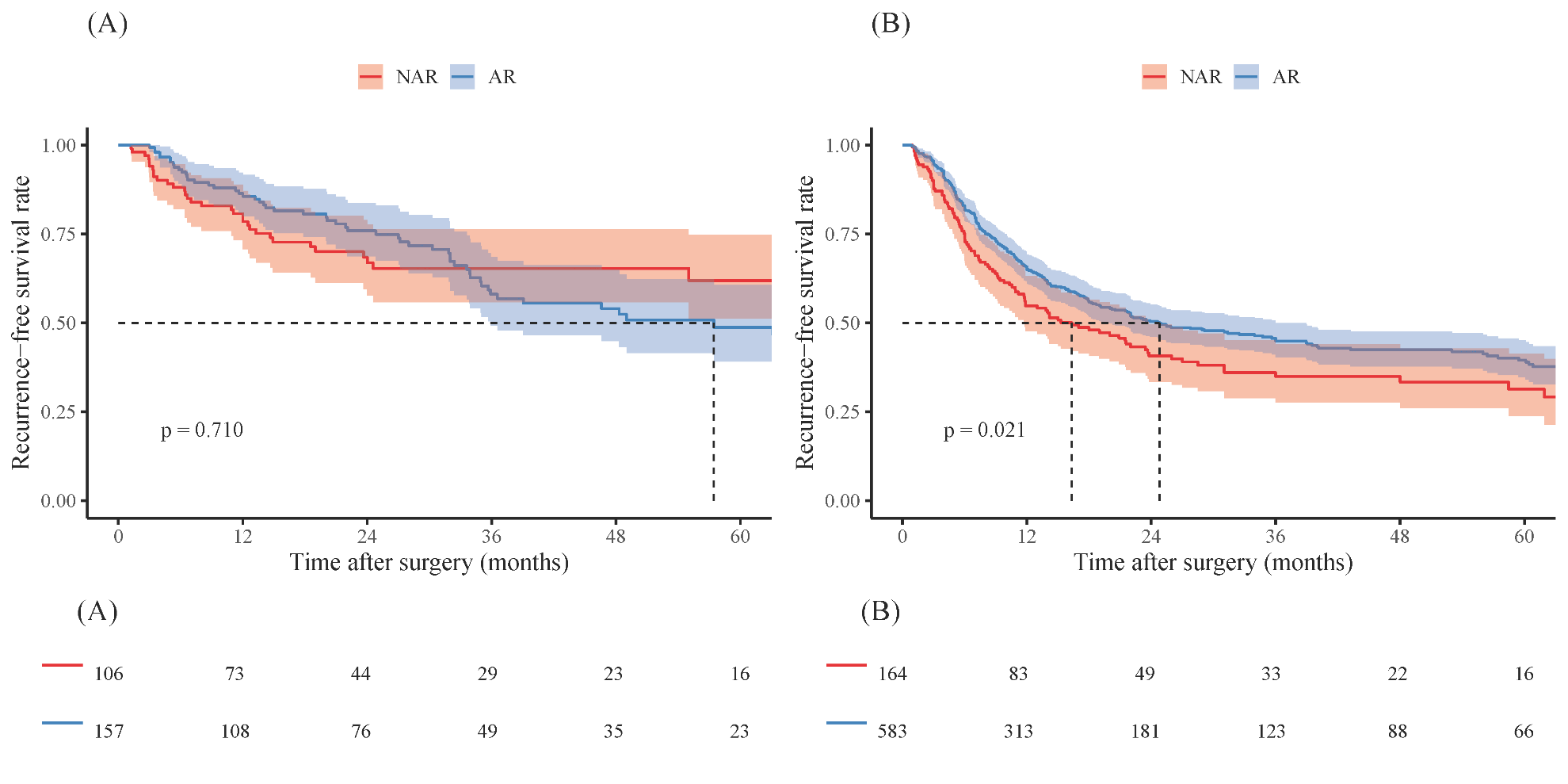


**Supplementary Table 1**. Comparison of patient characteristics between the two time periods (2000-2010 vs. 2011-2023).

| Characteristics | 2000-2010 | 2011-2023 | *P* value |
| --- | --- | --- | --- |
|  | n = 340 (35.1%) | n = 629 (64.9%) |  |
| Age (years) | 56 [49, 66] | 62 [54, 70] | < 0.001 |
| Sex, male | 199 (58.5) | 350 (55.6) | 0.425 |
| ASA classification, > 2 | 120 (35.3) | 254 (40.4) | 0.138 |
| Region, Eastern countries | 151 (44.4) | 254 (40.4) | 0.252 |
| Neoadjuvant chemotherapy | 10 (2.9) | 37 (5.9) | 0.060 |
| Cirrhosis | 84 (24.7) | 65 (10.3) | < 0.001 |
| ALBI score | -2.87 [-3.09, -2.59] | -2.98 [-3.24, -2.65] | 0.003 |
| CA19-9 (U/mL) | 43.8 [12.1, 182.4] | 32.0 [12.0, 111.5] | 0.168 |
| AR | 233 (68.5) | 473 (75.2) | 0.031 |
| Major hepatectomy | 168 (49.4) | 344 (54.7) | 0.133 |
| Minimally invasive surgery | 2 (0.6) | 44 (7.0) | < 0.001 |
| Lymphadenectomy | 132 (38.8) | 353 (56.1) | < 0.001 |
| Tumor size (cm) | 5.7 [4.0, 8.0] | 5.3 [3.5, 7.5] | 0.084 |
| Pathological T category |  |  | < 0.001 |
| T1 | 247 (72.6) | 393 (62.5) |  |
| T2 | 12 (3.5) | 129 (20.5) |  |
| T3 | 81 (23.8) | 107 (17.0) |  |
| Pathological N category |  |  | < 0.001 |
| N0 | 98 (28.8) | 224 (35.6) |  |
| N1 | 34 (10.0) | 129 (20.5) |  |
| Nx | 208 (61.2) | 276 (43.9) |  |
| Pathological TNM stage |  |  | < 0.001 |
| I | 229 (67.4) | 351 (55.8) |  |
| II | 11 (3.2) | 89 (14.1) |  |
| IIIA | 66 (19.4) | 60 (9.5) |  |
| IIIB | 34 (10.0) | 129 (20.5) |  |
| Surgical margin (mm) | 6.0 [3.0, 10.0] | 5.0 [2.0, 10.0] | 0.010 |
| Surgical margin, < 5.0 mm | 145 (42.6) | 299 (47.5) | 0.165 |
| Microvascular invasion | 63 (18.5) | 196 (31.2) | < 0.001 |
| Morphologic type, PI/MF+PI | 32 (9.4) | 60 (9.5) | 1.000 |
| Grade, poor/undifferentiated | 47 (13.8) | 102 (16.2) | 0.372 |
| Perineural invasion | 35 (10.3) | 130 (20.7) | < 0.001 |
| Severe complication | 35 (10.3) | 106 (16.9) | 0.008 |
| Adjuvant chemotherapy | 98 (28.8) | 173 (27.5) | 0.718 |

Data are presented as median (IQR) for continuous measures and n (%) for categorical measures.

Abbreviations: **ASA,** American society of Anesthesiologists; **ALBI,** albumin-bilirubin; **CA19-9,** Carbohydrate antigen; **AR,** anatomic resection; **PI/MF+PI,** periductal infiltrating/ mass forming plus periductal infiltrating

**Supplementary Table 2**. Multivariable COX regression analyses for recurrence stratified by tumor size of 4.0 cm.

|  |  | Tumor size < 4.0 cm | |  | Tumor size ≥ 4.0 cm | |
| --- | --- | --- | --- | --- | --- | --- |
| Variables | Reference | HR 95%CI | *P* value |  | HR 95%CI | *P* value |
| Age |  | 0.99 [0.97, 1.01] | 0.217 |  | 0.98 [0.98, 0.99] | **0.001** |
| Year of surgery, 2011-2023 | 2000-2010 | 0.85 [0.70, 1.02] | 0.492 |  | 0.81 [0.65, 1.01] | 0.066 |
| Cirrhosis |  | 1.43 [0.83, 2.46] | 0.203 |  | 1.27 [0.96, 1.69] | 0.095 |
| Tumor size |  | 1.01 [0.75, 1.35] | 0.964 |  | 1.07 [1.04, 1.11] | **< 0.001** |
| AR | NAR | 0.97 [0.59, 1.60] | 0.898 |  | 0.69 [0.54, 0.89] | **0.004** |
| Pathological T3 | T1 / T2 | 1.04 [0.57, 1.89] | 0.897 |  | 1.32 [1.00, 1.73] | 0.050 |
| Pathological N category |  |  |  |  |  |  |
| N1 | N0 | 1.74 [0.91, 3.31] | 0.094 |  | 1.61 [1.18, 2.20] | **0.003** |
| Nx | N0 | 1.08 [0.63, 1.85] | 0.773 |  | 1.16 [0.90, 1.50] | 0.239 |
| Microvascular invasion |  | 1.54 [0.94, 2.53] | 0.088 |  | 1.35 [1.03, 1.77] | **0.030** |
| Grade, poor/undifferentiated | Well/moderate | 1.49 [0.80, 2.79] | 0.207 |  | 1.11 [0.85, 1.46] | 0.433 |
| Perineural invasion | Minor hepatectomy | 1.49 [0.86, 2.58] | 0.160 |  | 1.18 [0.87, 1.61] | 0.277 |

Abbreviations: Carbohydrate antigen; **NAR,** non-anatomic resection; **AR,** anatomic resection; **PI/MF+PI,** periductal infiltrating/ mass forming plus periductal infiltrating; Bold font signify *P* value < 0.05

**Supplementary Table 3**. Comparison of recurrence patterns among 93 patients with tumors < 4 cm who experienced recurrence.

| Characteristics | NAR | AR | *P* value |
| --- | --- | --- | --- |
|  | 33 (35.5) | 60 (64.5) |  |
| Intrahepatic only | 21 (63.6) | 24 (40.0) | 0.076 |
| Intrahepatic and extrahepatic | 9 (27.3) | 18 (30.0) |  |
| Extrahepatic only | 2 (6.1) | 15 (25.0) |  |
| Unknown | 1 (3.0) | 3 (5.0) |  |

Data are presented as n (%) for categorical measures.

Abbreviations: **NAR,** non-anatomic resection; **AR,** anatomic resection

**Supplementary Table 4**. Comparison of the recurrence pattern among 377 patients with tumors ≥ 4.0 cm who experienced recurrence.

| Characteristics | NAR | AR | *P* value |
| --- | --- | --- | --- |
|  | n = 100 | n = 277 |  |
| Intrahepatic only | 74 (74.0) | 135 (48.7) | < 0.001 |
| Intrahepatic and extrahepatic | 7 (7.0) | 66 (23.8) |  |
| Extrahepatic only | 17 (17.0) | 70 (25.3) |  |
| Unknown | 2 (2.0) | 6 (2.2) |  |

Data are presented as n (%) for categorical measures.

Abbreviations: **NAR,** non-anatomic resection; **AR,** anatomic resection
